# Treatment of hypotrichosis simplex of the scalp with the combination of botanic extracts and minoxidil: a case report

**DOI:** 10.3389/fgene.2024.1491870

**Published:** 2025-01-20

**Authors:** Mingyue Zhuang, Yonglong Xu, Shiyi Zhong, Ziyuan Tian, Qingwu Liu, Dingquan Yang

**Affiliations:** ^1^ School of Clinical Medicine, Beijing University of Chinese Medicine, Beijing, China; ^2^ Department of Dermatology, China-Japan Friendship Hospital, Beijing, China

**Keywords:** botanical extracts, hypotrichosis simplex of the scalp, hair disease, family genealogy, genetic mutation

## Abstract

Hypotrichosis simplex of the scalp (HSS) is a clinically rare monogenic autosomal dominant disorder associated with variants in the gene *CDSN*, which encodes the desmosome protein corneodesmosin. Although studies have reported that some medications can improve the symptoms of hair loss in HSS, there is still a lack of definitive and effective treatments for this disease. We report a familial case of HSS in an 8-year-old male child diagnosed with HSS caused by a mutation in *CDSN*, who was treated with botanical extracts in combination with minoxidil, which resulted in significant hair growth after two treatments. This is the first study describing the improvement of clinical symptoms of HSS with oral botanical extracts. This suggests that botanical extracts in combination with minoxidil may be a therapeutic approach for HSS in the clinic.

## 1 Introduction

Hypotrichosis simplex of the scalp is a clinically low-morbidity monogenic autosomal dominant disorder characterized by progressive alopecia confined to the scalp (Levy-Nissenbaum et al., 2003). Affected individuals have normal hair from birth to infancy, but hair loss begins at 5–6 years of age and continues to worsen to almost complete baldness by 20–30 years of age. Other body hair including eyebrows, eyelashes, armpit hair, pubic hair and nails are unaffected. We report a case of 8 years old boy with increased hair loss and hair thinning since 1 year ago successfully treated with botanical extract which is first of its kind as per our knowledge.

## 2 Case presentation

**FIGURE 1:**
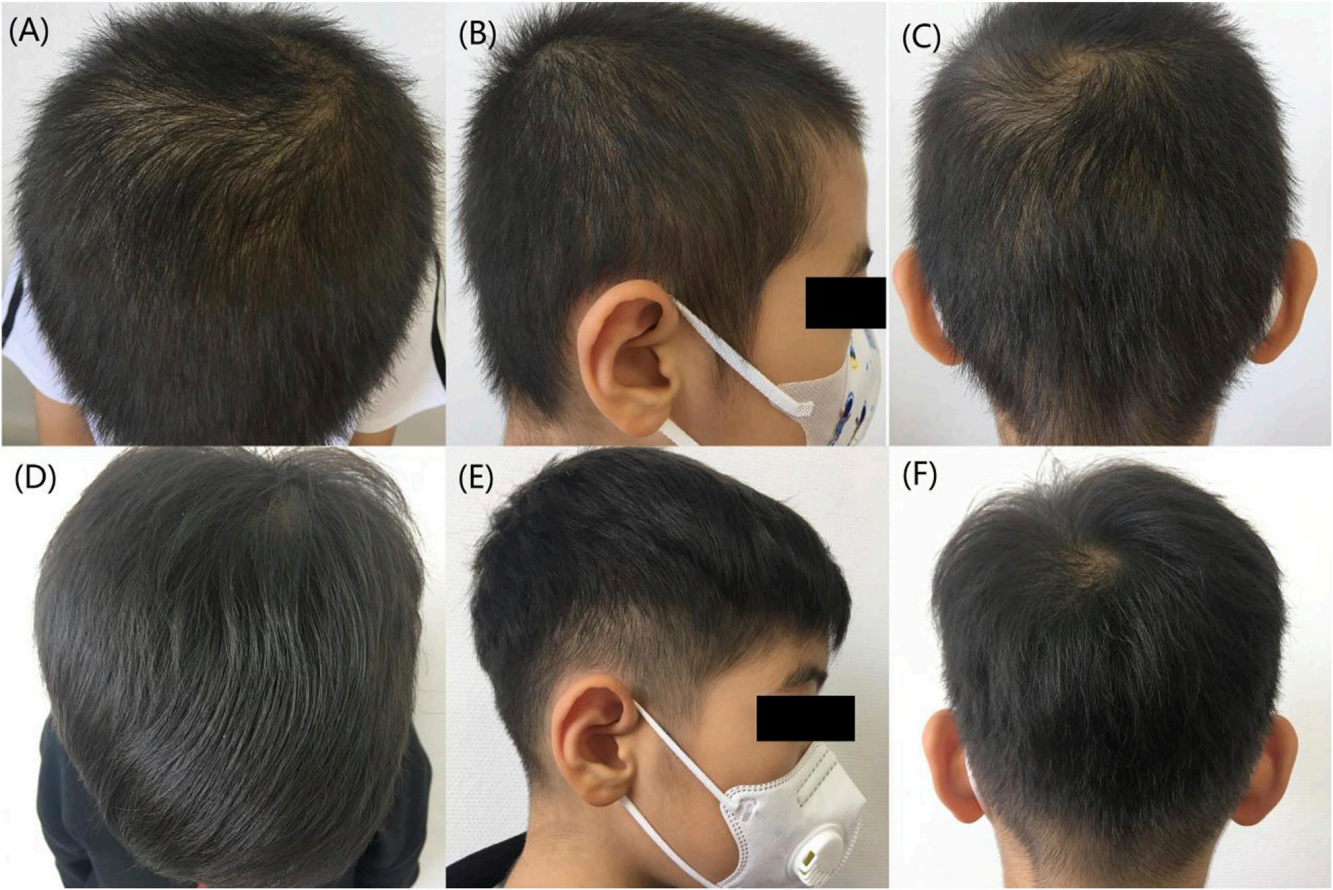
Comparison of hair before and after treatment. **(A–C)** Before treatment, the patient’s hair was thinning. **(D–F)** 6 months after treatment, a notable improvement in the patient’s hair condition was observed.

An 8-year-old boy presented to our outpatient clinic on 22 August 2022 for consultation. His hair was normal at birth, but he began to experience increased hair loss and hair thinning from the age of 7 years. Over the past year, the alopecia has continued to worsen leading to hair loss throughout the scalp. Dermatologic examination showed thinning and fine hair that were easily pulled out, no loss of eyebrows and eyelashes, no abnormalities in toenails and fingernails (Figures 1A–C). We performed scalp electron microscopy on the patient and found hair follicle openings suggests that the hair follicles are not shrinking. And can grow hair again. There is no terminal hair but there are fine vellus hairs. His parents and grandparents were assessed in our outpatient clinic. His mother and maternal grandfather were both affected from birth. The observation of hair thinning at birth was based on the family’s description. Although we did not have access to photographs from that time, the consistency of the family’s report and the subsequent progression of the condition in these individuals support the claim. The condition manifests itself from birth as thinning of hair, with no involvement of body hair such as eyelashes and eyebrows, and progresses gradually to total baldness. The proband’s father and maternal grandmother had normal hair.

**FIGURE 2:**
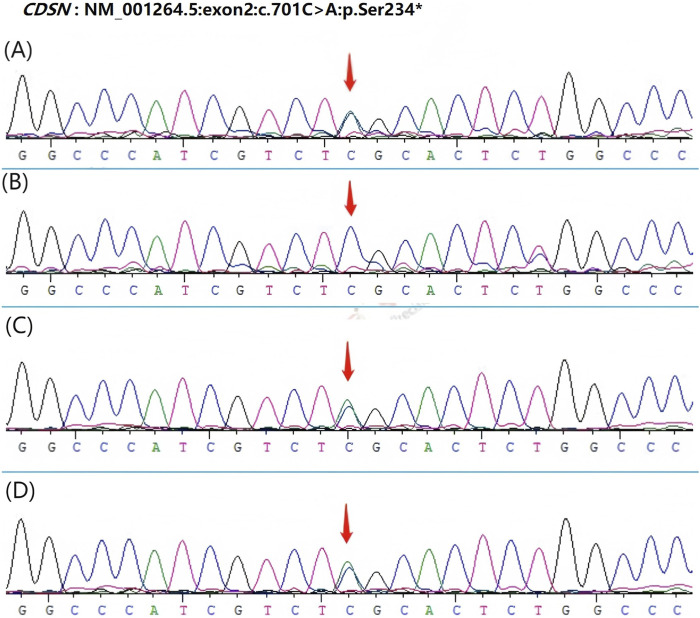
Sanger sequencing results of the *CDSN* gene in the patient and his relatives. The mutation c.701C > A is indicated by an arrow. **(A)** patient has heterozygous mutation in *CDSN* gene c.701C > A (p.Ser234*). **(B)** His father’s *CDSN* gene is wild-type. **(C–D)** H is mother and maternal grandfather carried the same heterozygous mutation in *CDSN* gene c.701C > A (p.Ser234*).

Based on the characteristics of the patient’s clinical presentation and the family history of hair loss, *CDSN* was selected as a candidate gene for whole exome sequencing. Whole exome sequencing of peripheral blood extracted from the patient and his relatives revealed a heterozygous mutation c.701C > A in the *CDSN* gene. This mutation results in a predicted change in the amino acid sequence of the corneodesmosin protein, which may affect its structure and function related to cell adhesion in the hair follicle. To further confirm this mutation, Sanger sequencing was carried out, and the results showed that the patient, his mother, and maternal grandfather carry the same heterozygous mutation in the *CDSN* gene, whereas his father and maternal grandmother have the wild-type *CDSN* gene (Figure 2).

Based on the characteristics of the patient’s clinical presentation and the results of family genetic testing, we diagnosed him with HSS. The patient used hair growth tincture (Z20053815, 60 mL/bottle; China-Japan Friendship Hospital, Beijing, China), 5 mL each time, once a day,Minoxidil (H20010714, 60 m/bottle, Zhejiang Wansheng Pharmaceutical Co., Ltd., China.), 1 mL each time, once a day, applied externally to the area of hair loss. Topical application of Minoxidil may cause irritating inflammation of the scalp such as itching and increased dandruff (Rossi et al., 2012). To prevent the above adverse reactions during administration, 10 mL of Hasinide solution (H34020418, 10 mL/bottle, Qiancheng Pharmaceutical Co., Ltd. Xuancheng, China.) is added to 60 mL of Minoxidil tincture to dilute and mix for use. The patient’s hair loss symptoms improved significantly after 4 months of treatment. The patient infected with COVID-19 causing exacerbation of hair loss in January 2023, he revisited our outpatient clinic to adjust his medication on 2 March 2023. Addition of oral compound glycyrrhizin tablets (J20130077, 110 mg per tablet; Minophagen Pharmaceutical Co., Ltd., Tokyo, Japan) 110 mg each time, twice daily. The patient’s liver and renal function have been monitored by regular follow-ups and the treatment has continued to the present day. The patient has returned to essentially the same level of hair as before the COVID-19 infection after 6 months (Figures 1D–F). In addition, the patient evaluated his hair growth according to the GPR (Global photographic review) and considered that there was a significant improvement from baseline at the six-month follow-up visit (70% improvement over baseline hairs).

## 3 Discussion

The *CDSN* gene is responsible for encoding the desmosome protein corneodesmosin, a highly differentiated epidermal glycoprotein expressed in the epidermis, the sclerotial epithelium, and the inner hair root sheath of the hair follicle. The desmosome proteins are strong cellular structures that connect neighbouring cells. Its structural disruption can cause keratin-forming cells to separate from each other, and abnormalities in desmosome proteins can cause a range of inherited dermatological disorders. It has been shown that nonsense mutations in the *CDSN* lead to the formation of short chains and the gradual accumulation of amyloid aggregates of corneodesmosin composed of normal *CDSN* genes, which may be toxic during hair growth, and are thus involved in the pathogenic mechanisms of hypotrichosis simplex of the scalp (HSS). The formation of short chains and the gradual accumulation of amyloid aggregates of desmosome proteins composed of normal *CDSN* genes, which may be toxic during hair growth, and are thus involved in the pathogenic mechanisms of hypotrichosis simplex of the scalp (HSS) (Caubet et al., 2010). In addition, desmosome protein has the function of cell adhesion, and mutations or genetic deletions of *CDSN* lead to peeling skin disease (PSD) (Valentin et al., 2015), which is clinically characterized by recurrent episodes of congenital scaly erythroderma with superficial skin shedding. In this paper, we report that both the patient and the family heterozygous gene carriers showed only simple hair loss without skin abnormalities such as itching and flaking. It may be due to the fact that the family pedigree in this case is characterized by a single mutation in *CDSN*, whereas all known PSD-causing *CDSN* mutations are either homozygous or compound heterozygous (Oji et al., 2010; Israeli et al., 2011; Mazereeuw-Hautier et al., 2011; Telem et al., 2012; Mallet et al., 2013; Kawakami et al., 2014), and the mode of inheritance is autosomal recessive, which rationally explains the patient’s manifestation of hypohidrosis without observing any abnormal skin manifestations.

The patient had normal hair at birth and began to lose hair by childhood, consistent with the classic presentation of hypotrichosis simplex of the scalp (HSS).In contrast, his mother and maternal grandfather showed hair thinning at birth although they carry the same heterozygous *CDSN* gene as the affected child. Therefore, we hypothesised that *CDSN* pathogenicity is characterised by individual-specific expression. The individual-specific expression might be related to factors such as epigenetic modifications or other genetic modifiers that could interact with the *CDSN* mutation.

There are no definitive treatment options for HSS, and conventional medications for alopecia areata have been clinically proven to be effective in treating HSS. The study reported that patients with HSS who received 3 months of oral treatment with growth factors (growth factors contain mainly hyaluronic acid and caffeine) and minoxidil showed significant improvements in hair thickness and density in patients compared to baseline (Vastarella et al., 2022). Regenerative therapies are also used in the treatment of HSS. Platelet-rich plasma (PRP) increases follicular and perifollicular angiogenesis by increasing platelet-derived growth factor (PDGF) and vascular endothelial growth factor (VEGF) levels,and Scalp injections of PRP combined with topical minoxidil therapy also achieved good results (Ramadan et al., 2023). In addition, gentamicin has gradually shown promise in the field of hereditary dermatoses caused by genetic mutations in recent years. Gentamicin was found to induce read-through activity of nonsense mutations causing HSS and alter the translation of corneodesmosin by primary keratinocytes in a patient with HSS (Peled et al., 2020), reducing the hypotrichosis phenotype.

In this case, the child was treated with minoxidil in combination with botanical extracts. Hair growth tincture is a herbal topical preparation of Psoraleae Fructus, Rhododendri Mollis flos and Zingiberis Rhizoma recens in 75% ethanol. It is a topical medication used to treat many types of hair loss,and it is made from mild ingredients with fewer side effects than minoxidil as a botanical extract preparation. The main ingredient of compound glycyrrhizin tablets is glycyrrhizin, an extract of liquorice (Yang et al., 2013). Compound glycyrrhizin tablets have anti-inflammatory, antiallergic, steroid, anticomplementary activity, and immunoregulation effects. It is commonly used to treat mild to moderate alopecia areata, due to the function of inhibiting the CD4 and CD8T cells and the cytokine generated by CD4 and CD8T cells (Yang et al., 2012). It can reduce inflammation-induced damage to the hair follicle growth cycle, restore the normal growth cycle of hair follicles, and promote hair growth. This helps to control the disease, restore hair growth and prevent further recurrence. Our team has used a regimen of oral compound glycyrrhizin tablets to treat severe alopecia areata in children and achieved safe and satisfactory treatment effects. We also verified that licorice extracts affect the pathogenesis of alopecia areata through the PI3K/AKT signalling pathway based on animal experiments (Lv et al., 2022). Minoxidil is a common medication for hair loss, but it often causes adverse reactions such as greasiness, itching, and increased dandruff on the scalp. The glucocorticoid Hasinide can alleviate the aforementioned side effects of Minoxidil and improve the follicular environment of the scalp.

Congenital absence or deficiency of hair can be a feature of many hereditary syndromes. It may develop alone or as a manifestation of a syndrome combined with involvement of other tissues and organs. More than 200 syndromic and 20 non-syndromic forms of congenital alopecia/hypospadias have been reported (Hayashi and Shimomura, 2022), HSS has its onset in childhood and presents only as thinning hairs, with no specific hair morphology or stem abnormalities, and the hair follicles may show miniaturised changes. Other body hair such as beards, eyebrows, eyelashes, armpit hair and nails are not affected. There are no other organ system abnormalities. It is easily misdiagnosed clinically as diffuse alopecia areata and severe androgenetic alopecia, as well as being differentially diagnosed from other congenital hair disorders. Therefore, a detailed medical and family history should be taken by the physician at the time of the patient’s visit. For clinically suspected cases, genetic testing is then recommended.

## 4 Conclusion

There is currently no definitive and effective treatment for hypotrichosis simplex of the scalp, this paper shows that minoxidil in combination with botanical extracts significantly improves HSS caused by mutations in the *CDSN* gene. Our team through preliminary animal experiments verified that licorice extract is closely related to the PI3K/AKT signaling pathway, the study shows that the PI3K-Akt signaling pathway plays a crucial role in hair follicle regeneration. However, the above is only speculative evidence based on literature studies, the specific mechanisms of action needs to be further verified at the histopathological and molecular dimensions of the hair follicle.

## Data Availability

The original contributions presented in the study are included in the article/Supplementary Material, further inquiries can be directed to the corresponding author.
