# Supplementary material for: Treatment of hypotrichosis simplex of the scalp with the combination of botanic extracts and minoxidil: a case report
Source: Front Genet. 2025 Jan 20;15:1491870. doi: 10.3389/fgene.2024.1491870 (PMC11788402; doi:10.3389/fgene.2024.1491870)
Supplement: Supplementary file 1 [file DataSheet1.docx]

Supplementary Figures


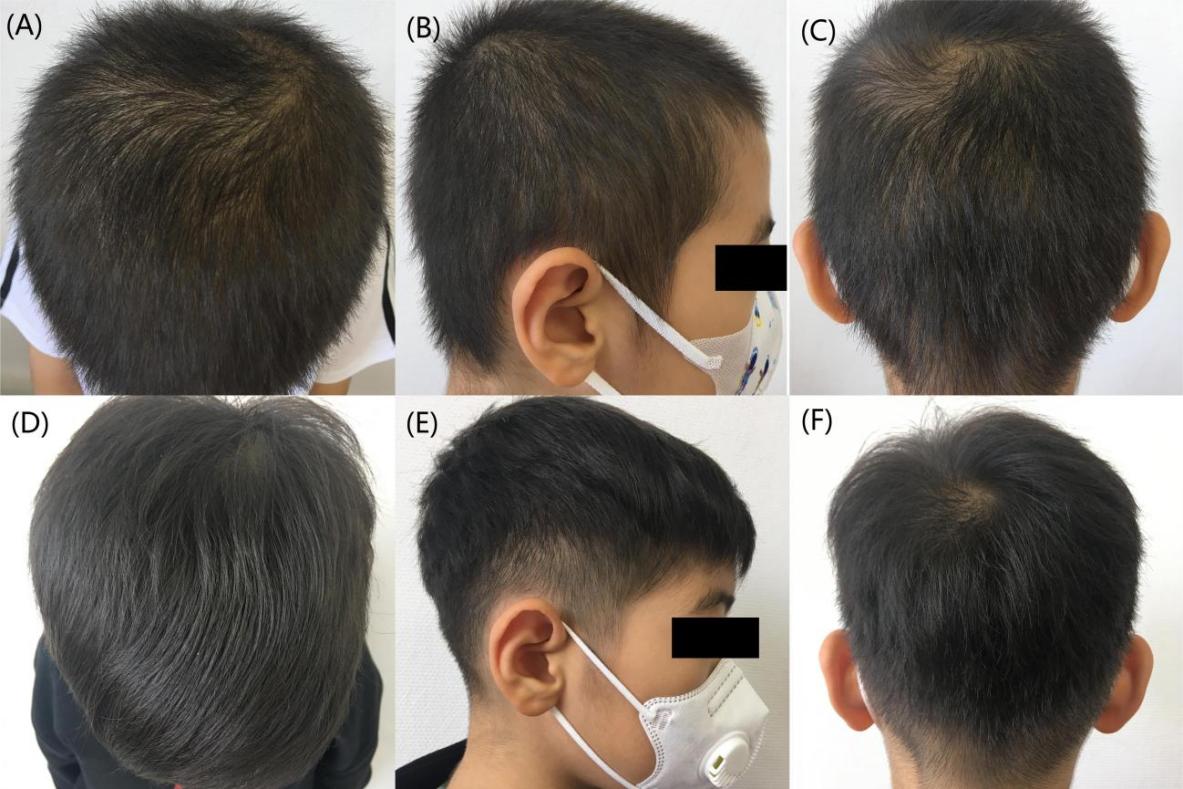


**Supplementary Figure 1.** Comparison of hair before and after treatment.(A-C)Before treatment, the patient's hair was thinning. (D-F) 6 months after treatment, a notable improvement in the patient's hair condition was observed.


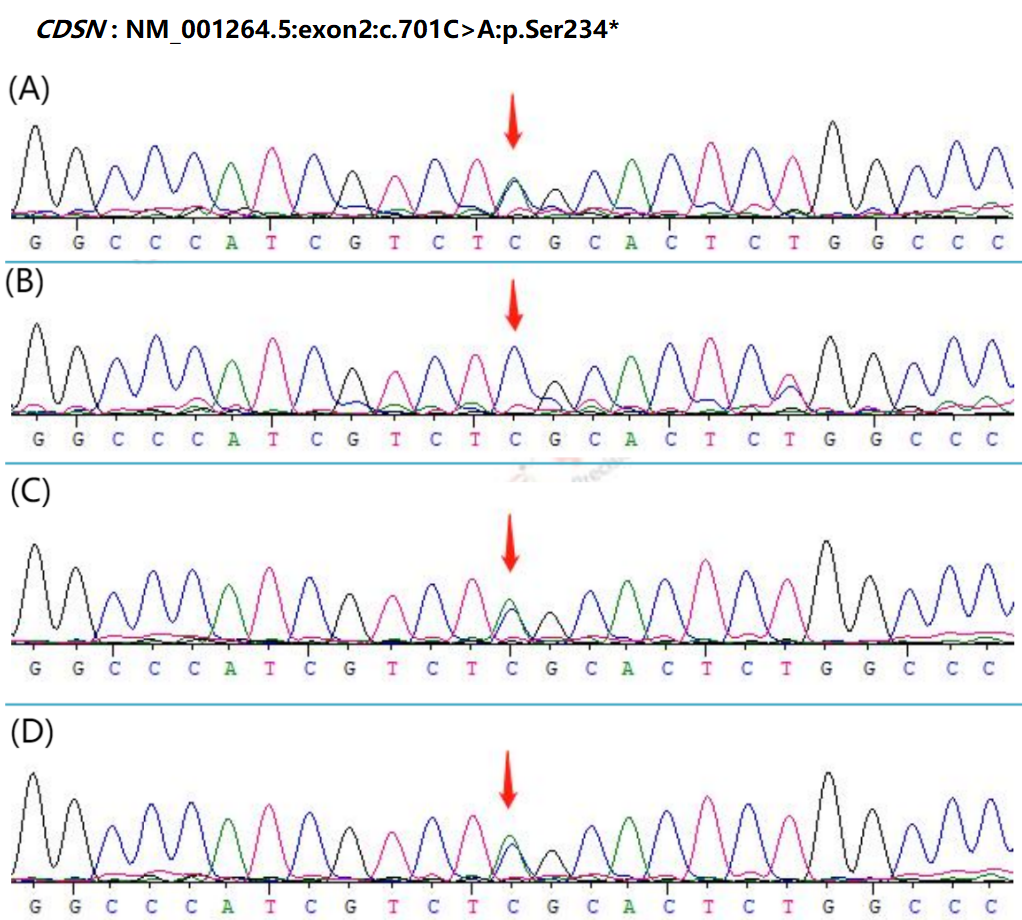


**Supplementary Figure 2.** Sanger sequencing results of the *CDSN* gene in the patient and his relatives. The mutation c.701C > A is indicated by an arrow. (A)patient has heterozygous mutation in *CDSN* gene c.701C>A(p.Ser234*).(B)His father's *CDSN* gene is wild-type.(C-D)His mother and maternal grandfather carried the same heterozygous mutation in *CDSN* gene c.701C>A(p.Ser234*).
